# Associations between fully-automated, 3D-based functional analysis of the left atrium and classification schemes in atrial fibrillation

**DOI:** 10.1371/journal.pone.0272011

**Published:** 2022-08-15

**Authors:** Maurice Pradella, Constantin Anastasopoulos, Shan Yang, Manuela Moor, Patrick Badertscher, Julian E. Gehweiler, Florian Spies, Philip Haaf, Michael Zellweger, Gregor Sommer, Bram Stieltjes, Jens Bremerich, Stefan Osswald, Michael Kühne, Christian Sticherling, Sven Knecht

**Affiliations:** 1 Department of Radiology, University Hospital Basel, University of Basel, Basel, Switzerland; 2 Department of Radiology, Northwestern University Feinberg School of Medicine, Chicago, Illinois, United States of America; 3 Department of Cardiology/Electrophysiology, University Hospital Basel, University of Basel, Basel, Switzerland; 4 Cardiovascular Research Institute Basel (CRIB), Basel, Switzerland; 5 Hirslanden Klinik St. Anna, Luzern, Switzerland; Scuola Superiore Sant’Anna, ITALY

## Abstract

**Background:**

Atrial fibrillation (AF) has been linked to left atrial (LA) enlargement. Whereas most studies focused on 2D-based estimation of static LA volume (LAV), we used a fully-automatic convolutional neural network (CNN) for time-resolved (CINE) volumetry of the whole LA on cardiac MRI (cMRI). Aim was to investigate associations between functional parameters from fully-automated, 3D-based analysis of the LA and current classification schemes in AF.

**Methods:**

We retrospectively analyzed consecutive AF patients who underwent cMRI on 1.5T systems including a stack of oblique-axial CINE series covering the whole LA. The LA was automatically segmented by a validated CNN. In the resulting volume-time curves, maximum, minimum and LAV before atrial contraction were automatically identified. Active, passive and total LA emptying fractions (LAEF) were calculated and compared to clinical classifications (AF Burden score (AFBS), increased stroke risk (CHA_2_DS_2_VASc≥2), AF type (paroxysmal/persistent), EHRA score, and AF risk factors). Moreover, multivariable linear regression models (mLRM) were used to identify associations with AF risk factors.

**Results:**

Overall, 102 patients (age 61±9 years, 17% female) were analyzed. Active LAEF (LAEF_active) decreased significantly with an increase of AFBS (minimal: 44.0%, mild: 36.2%, moderate: 31.7%, severe: 20.8%, p<0.003) which was primarily caused by an increase of minimum LAV. Likewise, LAEF_active was lower in patients with increased stroke risk (30.7% vs. 38.9%, p = 0.002). AF type and EHRA score did not show significant differences between groups. In mLRM, a decrease of LAEF_active was associated with higher age (per year: -0.3%, p = 0.02), higher AFBS (per category: -4.2%, p<0.03) and heart failure (-12.1%, p<0.04).

**Conclusions:**

Fully-automatic morphometry of the whole LA derived from cMRI showed significant relationships between LAEF_active with increased stroke risk and severity of AFBS. Furthermore, higher age, higher AFBS and presence of heart failure were independent predictors of reduced LAEF_active, indicating its potential usefulness as an imaging biomarker.

## Introduction

Atrial fibrillation (AF) is the most common arrhythmic heart disease affecting about 1% of the general population and more than 12 million people in the US are expected to have AF by 2030 [1, 2]. This is of clinical importance due to the association of AF with an increased stroke risk, reduction of quality of life and cognitive decline [3–5]. Several risk factors are associated with the development of AF, including age, AF type, AF Burden and cardiovascular (CV) risk factors such as arterial hypertension (HT), diabetes mellitus, and heart failure (HF) [6]. Furthermore, secondary conditions like hyperthyroidism or lifestyle factors may precipitate AF [7, 8].

Multiple studies have shown that remodeling of the left atrium (LA) with AF leads to its enlargement [9–11]. However, LA enlargement might not present in some specific AF etiologies [12, 13]. In consequence, current guidelines recommend the assessment of LA size for AF patients, commonly based on the static dimension in parasternal long axis, LA volume (LAV), or indexed LAV (LAVi) [14]. Volumetric parameters are mainly calculated from 2-dimensional data using volumetric approximations such as the area-length method. Besides static LAV assessment, LA function is another important parameter for its characterization. It can be measured from time-resolved (CINE) imaging as strain or LA emptying fractions (LAEF) [15]. The latter was first established based on echocardiography. Recently, preserved LAEF was reported to predict the outcome after HF using cardiac MRI (cMRI) [16]. Moreover, LA function was identified as predictor of CV events and the outcome after myocardial infarction [17, 18]. However, LAEF assessment can be time consuming and requires specific knowledge to preprocess the imaging data.

Artificial intelligence (AI) and specifically deep learning (DL) have proven to support performing LA segmentations on single time point of the cardiac cycle or on post-contrast cMRI with excellent results [19, 20]. DL-based segmentation of the whole LA over the cardiac cycle based on CINE cMRI has also been validated recently for biplane and 3D-based assessment [21]. But this technique has not been applied in a patient cohort to investigate associations with clinical parameters.

The aims of this study were twofold. First, to assess the application of a fully-automatic approach to quantify LA functional parameters based on cMRI in a cohort of AF patients. Second, to investigate its associations with established and novel clinical classification schemes of AF and CV risk factors.

## Materials and methods

### Study population

The study was approved by the local ethics committee (Ethikkommission Nordwest- und Zentralschweiz (EKNZ)) and complied with the Declaration of Helsinki. Patients gave written informed consent. We considered 181 consecutive patients with CINE MRI from our prospective AF cohort (SWISS AF PVI, Clinical Trial registry) retrospectively. Exclusion criteria were any prior LA ablation and AF during the cMRI. In addition, we performed a comprehensive analysis on all patients, excluding only patients with prior LA ablation. From study records, we extracted CV risk factors (HF, HT, diabetes, renal failure) and left ventricular ejection fraction (LVEF, based on echocardiography).

### Image acquisition

cMRI scans were performed on 1.5T MRI systems (Siemens Avanto or Espree, Siemens Healthineers, Germany). Retrospectively ECG-gated, balanced steady-state free precision CINE sequences in oblique-axial orientation (planning was based on a 4CH scout) were acquired covering the whole LA in up to 12 axial stacks during breathhold (TE: 1.1–1.2ms, flip angle: 58–64°, in-plane resolution: 192x156mm, spatial resolution: 1.8–2.0 x 1.8–2.0mm, slice thickness: 6mm, no section gap) with 25 frames per cardiac cycle. No further long- or short-axis views were included in the study protocol.

### Convolutional neural network

**Fig 1:**
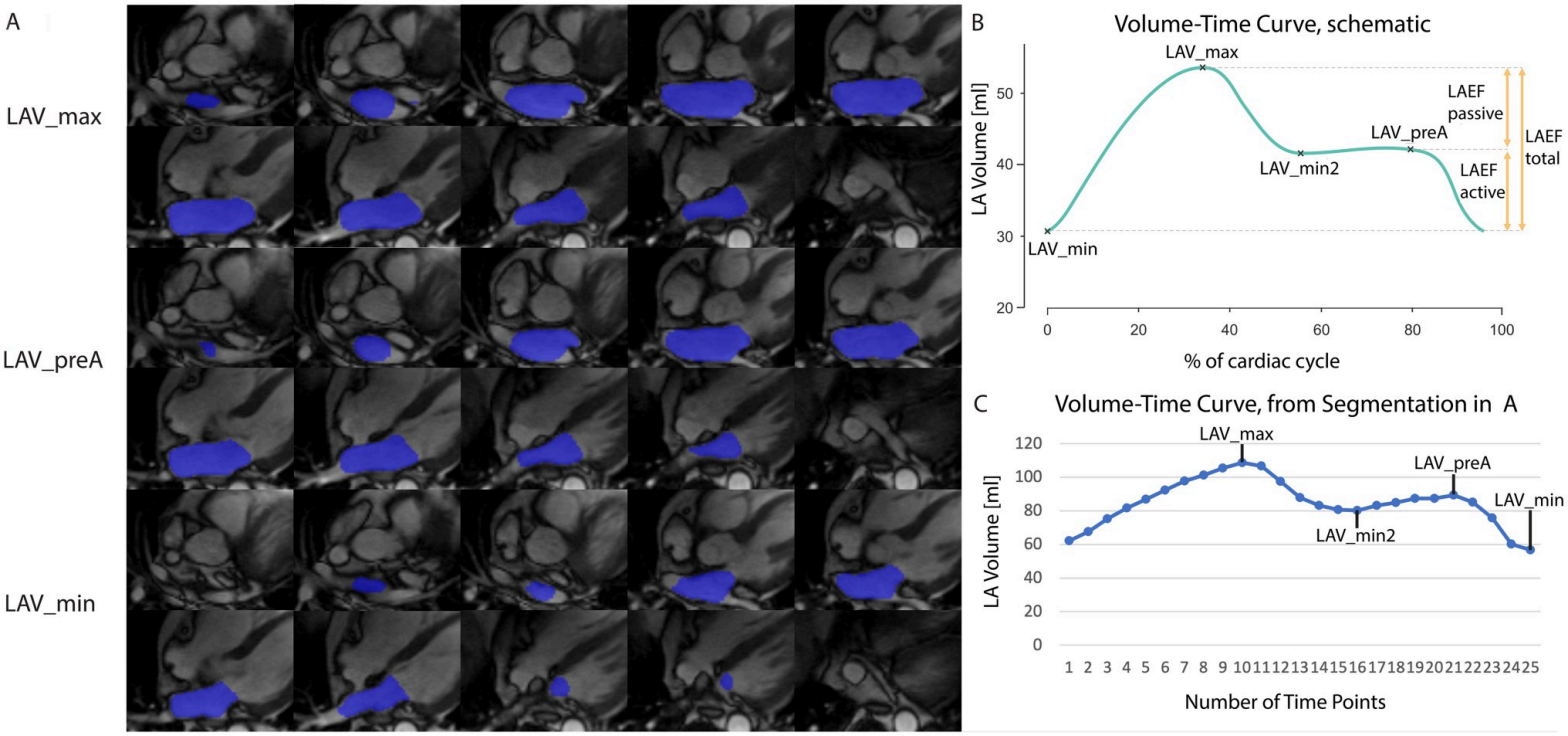
LA segmentation performed by the CNN. A) Example case of LA segmentation performed by the CNN on oblique-axial CINE images. Segmentations are shown at the time point of maximum volume (LAV_max, top), volume before atrial contraction (LAV_preA, center) and minimum volume (LAV_min, bottom). B) Schematic volume-time curve to demonstrate the respective fiducial points for minimum LA Volume (LAV_min), maximum LA Volume (LAV_max), volume before atrial contraction (LAV_preA) and local minimum between LAV_max and LAV_preA (LAV_min2). C) volume-time curve of the case shown in (A) with all fiducial points identified.

The network was described and validated in detail elsewhere [21]. It was built to segment the LA using the area-length method (2D) and on multiple oblique-axial CINE sequences in 4CH orientation (3D); the latter was used in this study. Briefly, manual segmentations were performed by M.P. and S.K. using the oblique-axial CINE stack covering the whole LA in 50 cases (Segment v2.2 R6435, Medviso, Sweden) [22]. These time-resolved segmentations were exported as binary masks, each with 25 images / time points, resulting in 1,250 volumes. These segmentations served as the training dataset for a deep convolutional neural network (CNN), based on a U-Net architecture [23]. An anisotropic, slightly altered version of the original 3D U-Net was implemented for the segmentation of the LA with three resolution layers formed by two pooling and upsampling layers [24]. After training and validation, the resulting network was used to predict the segmentation for all cases. An example case with segmentation at fiducial time points is shown in Fig 1.

### LA functional assessment from whole cardiac cycle

LA volume was calculated from the segmented 3D dataset by the sum of all identified LA voxels. This was performed for every time point of the cardiac cycle to create the volume-time curve for the LA (Fig 1). LAV_max and LAV_min were automatically identified. Additional fiducial points were the volume before atrial contraction (LAV_preA) which is defined as the time point just before the atrial contraction assists in emptying the LA [25]. Furthermore, the minimal volume between LAV_max and LAV_preA (LAV_min2) was identified automatically, primarily in order to guide the custom-written computational code (in MATLAB, MathWorks, USA) to perform automatic detection of fiducial points. If all fiducial points were identified, the patient was included in the main analysis. Based on available fiducial points, the total (LAEF_total), active (LAEF_active) and passive LAEF (LAEF_passive) were automatically calculated (Fig 1) [25, 26]:

$$LAEF_{total}(\%)=({LAV}_{max}\;-\;LAV_{min})\;÷{LAV}_{max}\;*100$$

$$LAEF_{active}(\%)=({LAV}_{preA}\;-\;LAV_{min})\;÷{LAV}_{preA}\;*100$$

$$LAEF_{passive}(\%)=({LAV}_{max}\;-\;LAV_{preA})\;÷{LAV}_{max}\;*100$$

Patients without the fiducial point of LAV_preA due to AF during MRI acquisition were included in the comprehensive analysis. In addition to the absolute volumes, we calculated the respective indexed LAV using the Body Surface Area (BSA) according to the Mosteller formula resulting in maximum (LAVi_max), minimum indexed LA volume (LAVi_min), indexed volume before atrial contraction (LAVi_preA), and indexed minimum volume between LAV_max and LAV_preA (LAVi_min2).

**Fig 2:**
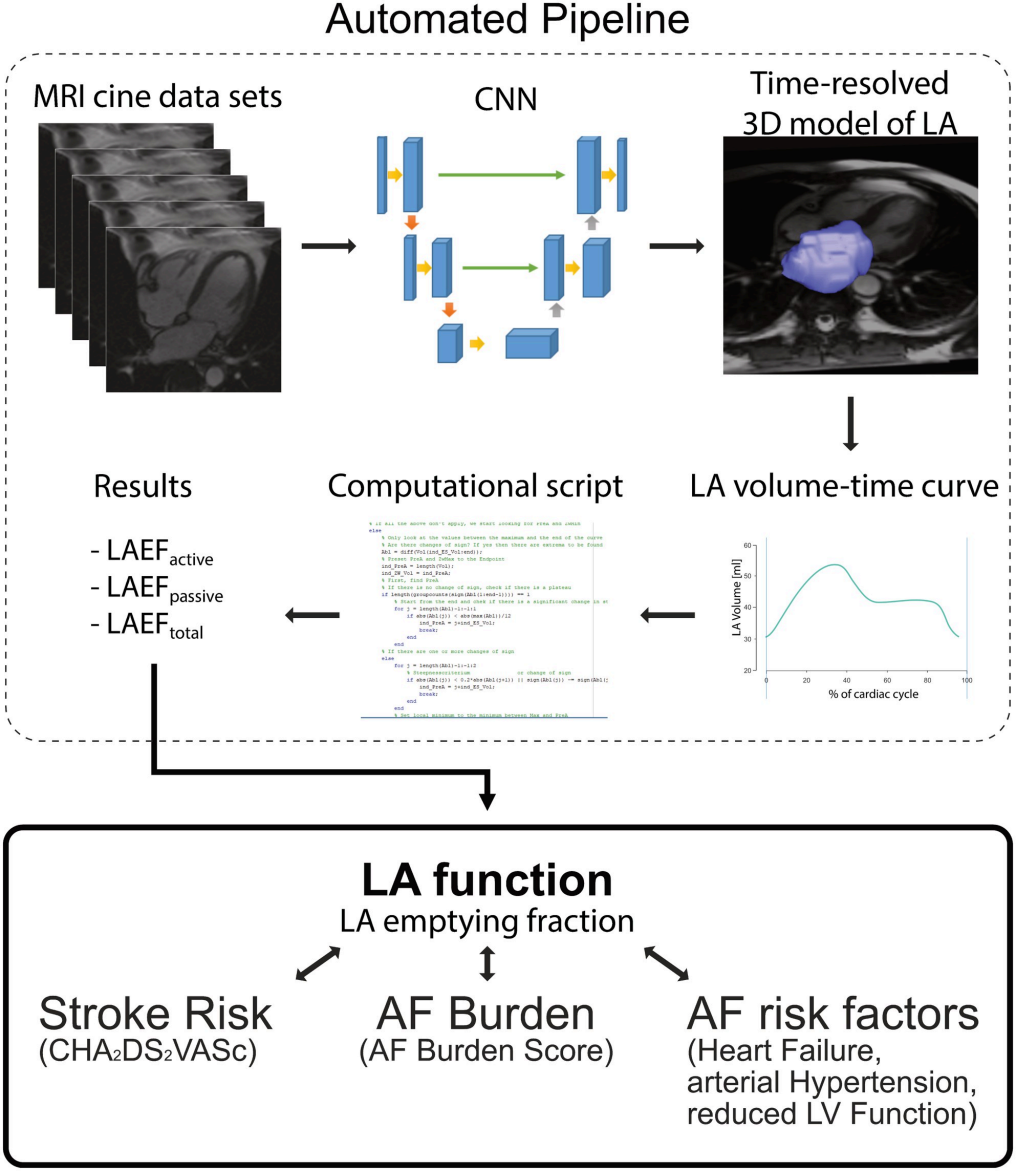
Overview of automatic workflow. The automated pipeline for image processing is shown in the dashed rectangle at the top: a stack of oblique axial CINE MRI series in 4CH orientation was processed by the convolutional neural network (CNN) for segmentation of the left atrium (LA). Based on the resulting time-resolved 3D model and LA volume-time curve, the characteristic time points were identified. The resulting functional LA parameters (LA emptying fractions (LAEF)) were available fully-automatic. Those parameters were investigated further in relation to stroke risk (CHA_2_DS_2_VASc), atrial fibrillation (AF) Burden (AF Burden Score) and other established AF risk factors.

An overview of the entire automatic workflow pipeline can be found in Fig 2.

### Classification of atrial fibrillation

Standard classification of AF was performed based on the presentation, duration and spontaneous termination of the AF episodes resulting in the class of paroxysmal, and persistent AF [14]. In the recently proposed “4S-AF” scheme, the stroke risk (based on the CHA_2_DS_2_VASc score), symptom severity (EHRA symptom score), and severity of AF Burden were proposed for the structured characterization of AF [14]. Stroke risk was stratified for low-risk (CHA_2_DS_2_VASc≤1) and increased risk (CHA_2_DS_2_VASc≥2). Classification of the EHRA score was performed as follows: class 1 (no symptoms), class 2 (mild symptoms), class 3 (severe symptoms), class 4 (disabling symptoms). The severity of AF was characterized based on the established classification of paroxysmal and persistent AF and additionally, using an established, symptomatic burden-based classification (AF Burden score (AFBS)). AFBS is a structured clinical assessment to evaluate frequency and duration of AF episodes as well as number of electrical cardioversions [27]. The sum of the frequency [daily (5 points), two or more days a week (4), once a week (3 points), monthly (2 points), <4 times per year (1 point)], the duration of the event [minutes (1 point), hours (2 points), most of the day (3), all day (4 points)], and the number of electrical cardioversions [1 (1 point), 2 (2 points), 3 (3 points), >3 (4 points)] was calculated and grouped as minimal (1–3 points), mild (4–6), moderate (7–9), and severe (≥10) AF Burden.

### Statistical analysis

Baseline characteristics of patients are presented as the count and percentage for categorical variables. For comparison of the continuous variables, Shapiro test for normality was performed, followed by either t-test (two groups) or one way ANOVA (more than two groups) in case of normal distribution. If data was not normally distributed, we performed Kruskal Wallis test. If there were more than two groups, posthoc Bonferroni correction was performed. Continuous variables were reported as mean ± standard deviation (SD) for normal distribution or median ± interquartile range (IQR) for non-normal distribution. Discrete variables were compared using Fisher’s exact test.

We used a multivariable linear regression model (mLRM) in a step-wise forward approach, corrected for age, BMI and sex, to investigate the relationship of functional LA parameters with following clinical parameters: AF type, AFBS, EHRA score, CHA_2_DS_2_VASc, diagnosis of HT, diabetes, HF, renal failure, and LVEF. Parameters with p-value < 0.1 were considered for the next step in the forward approach. Results of the univariable linear regression models (uLRM) are included in the Supplemental Material. Statistical analyses were performed using SPSS (IBM, USA) and a p-value < 0.05 was considered statistically significant.

## Results

### Study cohort

**Fig 3:**
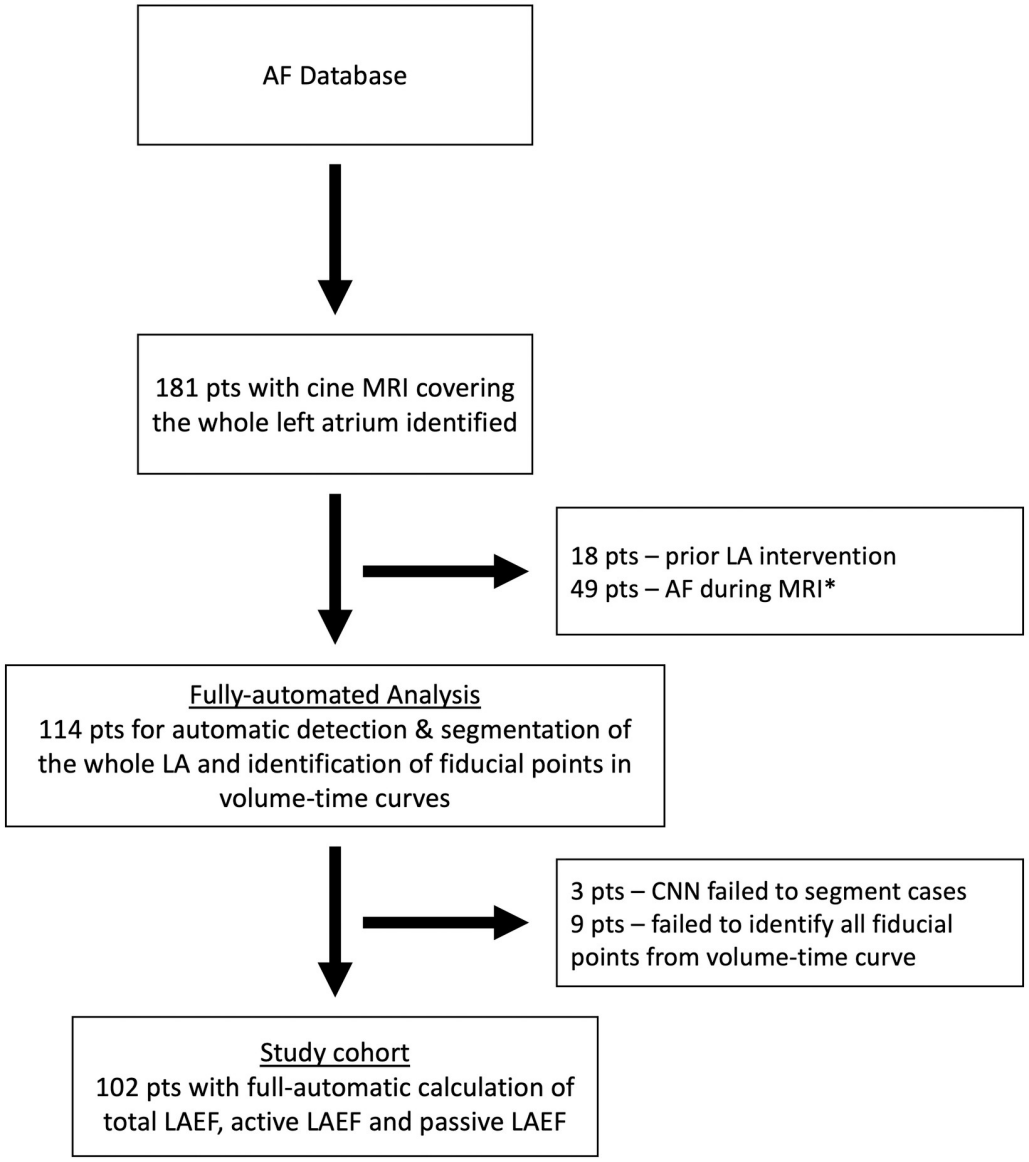
Study cohort flowchart. * We performed an additional comprehensive analysis of all patients independent from rhythm status during MRI (total n = 151, study cohort (n = 102) and patients with AF during MRI (n = 49)). However, assessment of active LA contraction was not possible in this cohort.

We finally analyzed 102 patients with automatically calculated LAEF_total, LAEF_active and LAEF_passive. The segmentation algorithm failed in three patients, in nine patients not all fiducial points could be identified due to multiple extra-systoles during MRI acquisition (Fig 3). The baseline characteristics can be found in Table 1.

**Table 1:** Baseline data.

	n = 102
Age [years]	60.8±8.9
Sex, female	18 (17.6%)
BMI [kg/m^2^]	26.8±4.0
BSA [m^2^]	2.02±0.21
Type AF	
Paroxysmal	73 (71.6%)
Persistent	29 (28.4%)
CV risk factors	
Arterial hypertension	55 (53.9%)
Heart failure	7 (6.9%)
Diabetes	7 (6.9%)
Renal failure	8 (7.8%)
Myocardial infarction	3 (2.9%)
Stroke	7 (6.9%)
Smoking status	
Never	41 (40.2%)
Active	11 (10.8%)
Former	50 (49.0%)
CHA_2_DS_2_VASc	
0	23 (22.5%)
1	31 (30.4%)
2	29 (28.4%)
3	11 (10.8%)
4	6 (5.9%)
5	1 (1.0%)
6	0
CHA_2_DS_2_VASc	
Low stroke risk (CHA_2_DS_2_VASc ≤ 1)	55 (53.9%)
Increased stroke risk (CHA_2_DS_2_VASc ≥ 2)	47 (46.1%)
EHRA score	
I	8 (8%)
II	57 (56%)
III	30 (29%)
IV	1 (1%)
AF Burden score (AFBS)	
1	8 (7.8%)
2	65 (63.7%)
3	23 (22.5%)
4	5 (4.9%)
LA parameters	
LAV_max [ml]	102.5±34.2
LAV_min [ml]	54.3±28.8
LAV_preA [ml]	80.8±30.9
LAV_min2 [ml]	76.3±30.5
LAVi_max [ml/m^2^]	50.9 ± 16.1
LAVi_min [ml/m^2^]	27.0 ± 14.3
LAVi_preA [ml/m^2^]	40.1±14.7
LAVi_min2 [ml/m^2^]	37.9±14.8
LAEF_total [%]	48.6±12.1
LAEF_active [%]	35.2±12.2
LAEF_passive [%]	21.7±7.9
LVEF [%]	57.4±8.0

### Association of functional parameters with stroke risk based on CHA_2_DS_2_VASc

**Fig 4:**
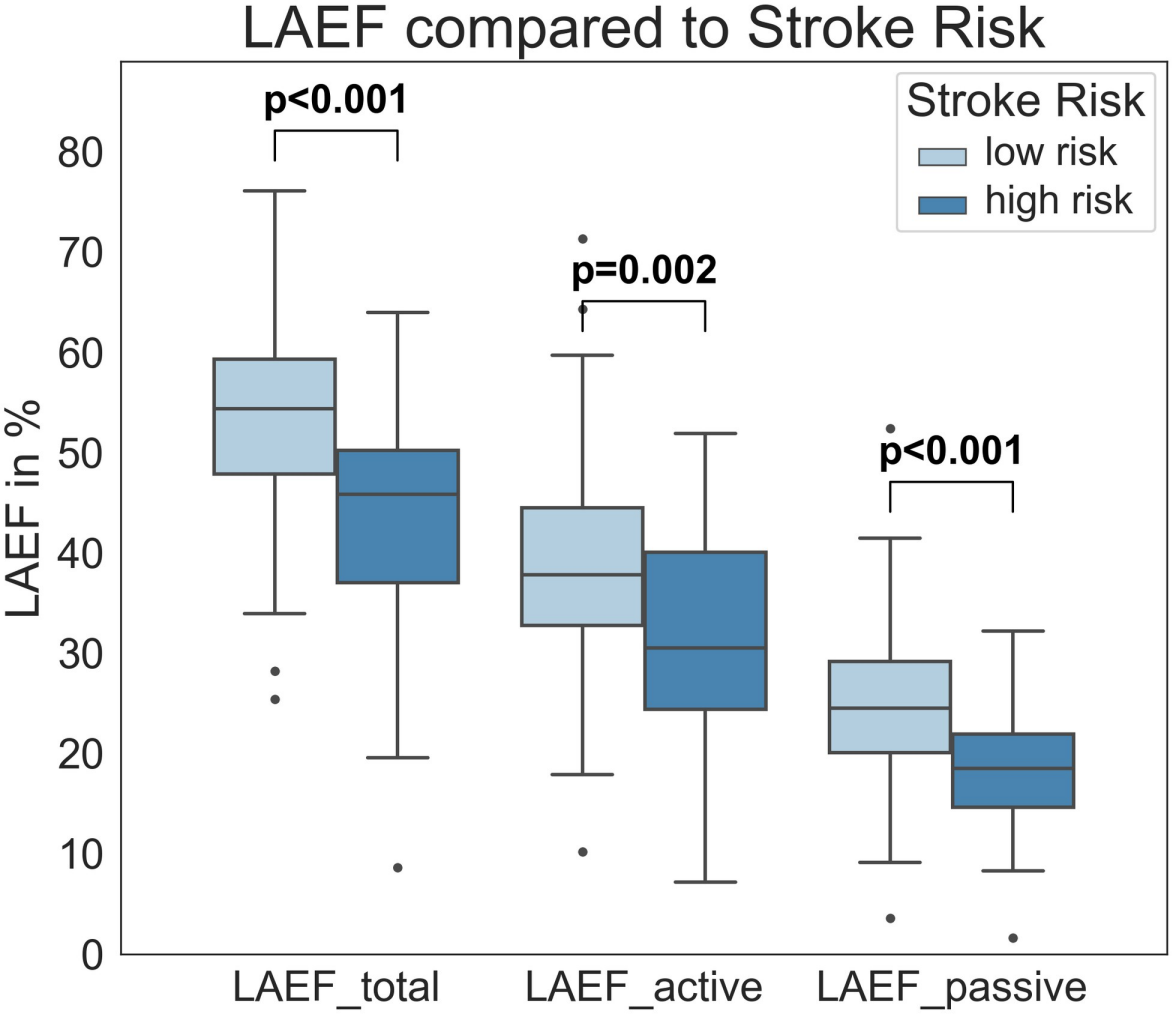
LA functional parameters and stroke risk based on CHA_2_DS_2_VASc. LAEF_total, LAEF_active and LAEF_passive in relation to low and increased stroke risk (based on CHA_2_DS_2_VASc score of ≤ 1 or ≥ 2). All three parameters were significantly lower in the group with increased stroke risk based on the CHA_2_DS_2_VASc. LAEF–left atrial emptying fraction.

The three functional parameters LAEF_total, LAEF_active, and LAEF_passive were all significantly lower for increased stroke risk (CHA_2_DS_2_VASc ≥2; p<0.001, p = 0.002 and p<0.001, respectively; Fig 4, Table 2). This was based upon significant increases in minimum LAV parameters (LAV_min, LAV_preA, LAVi_min, LAVi_preA). A detailed CHA_2_DS_2_VASc comparison for all categories can be found in the S1 Table.

**Table 2:** Functional associations with low and increased stroke risk based on CHA_2_DS_2_VASc.

	CHA_2_DS_2_VASc based stroke risk		
Stroke risk [patients]	Low (CHA_2_DS_2_VASc ≤ 1) [55]	Increased (CHA_2_DS_2_VASc ≥ 2) [47]	p value
LAV_max [ml], mean ± SD	99.3 ± 34.6	106.4 ± 33.6	0.30
LAV_min [ml], median ± IQR	42.9 ± 22.7	57.2 ± 34.7	**0.004**
LAV_preA [ml], median ± IQR	70.7 ± 33.2	83.0 ± 30.6	**0.01**
LAV_min2 [ml], median ± IQR	64.7 ± 28.8	81.4 ± 32.2	**0.003**
LAVi_max [ml/m^2^], mean ± SD	48.2 ± 14.4	54.1 ± 17.4	0.06
LAVi_min [ml/m^2^], median ± IQR	22.8 ± 9.1	31.9 ± 17.5	**0.002**
LAVi_preA [ml/m^2^], median ± IQR	35.5 ± 12.2	43.6 ± 16.7	**0.005**
LAVi_min2 [ml/m^2^], median ± IQR	32.1 ± 12.8	40.7 ± 17.7	**0.001**
LAEF_total [%], median ± IQR	54.4 ± 11.4	45.8 ± 13.2	**<0.001**
LAEF_active [%], median ± IQR	37.8 ± 11.7	30.5 ± 15.6	**0.002**
LAEF_passive [%], median ± IQR	24.5 ± 9.1	18.5 ± 7.3	**<0.001**

LAEF–Left atrial emptying fraction, LAV–Left atrial volume, LAVi–Left atrial volume index

### Association of functional parameters with severity of atrial fibrillation burden

**Fig 5:**
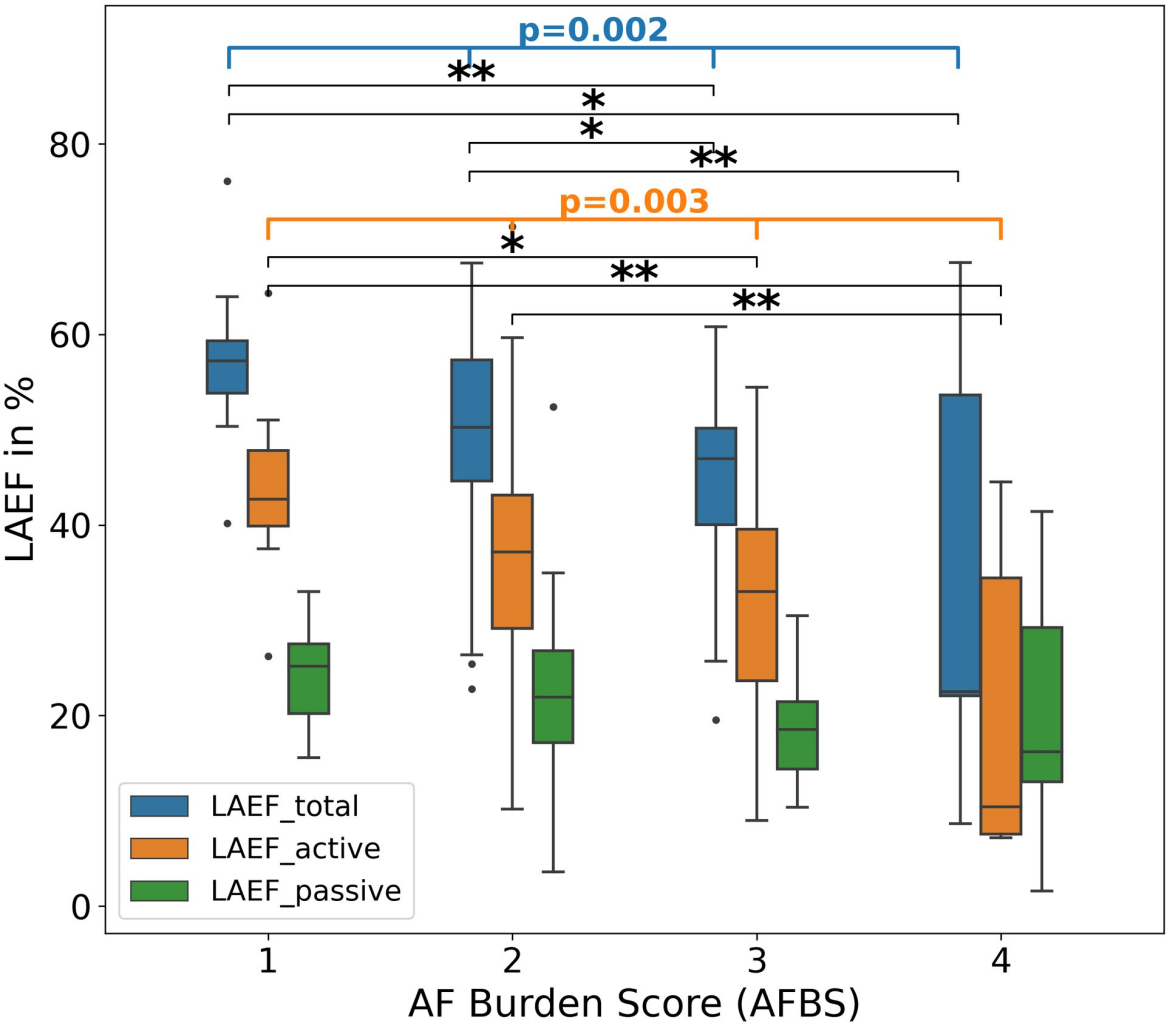
LA functional parameters and AFBS. LAEF_total, LAEF_active and LAEF_passive in relation to the AFBS categories. Both LAEF_total and LAEF_active significantly decreased with AFBS increase while LAEF_passive did not show significant differences. LAEF–left atrial emptying fraction. AFBS–AF Burden score. Post hoc tests: * = p<0.05, ** = p<0.01.

LAEF_total and LAEF_active significantly decreased with increase of AFBS (p = 0.002 and p = 0.003, respectively) (Table 3). For LAEF_total, a posthoc test revealed significant differences between AF Burden categories 1 vs. 3 (p = 0.007), 1 vs. 4 (p = 0.04), 2 vs. 3 (p = 0.04), and 2 vs. 4 (p = 0.007). For LAEF_active, significant differences were found between AF Burden categories 1 vs. 3 (p = 0.02), 1 vs. 4 (p = 0.01), and 2 vs. 4 (p = 0.005) (Fig 5). LAEF_passive was not significantly different between groups. This was mainly driven by significant increases of LAV_min, LAVi_min and LAVi_preA when AF Burden increase (p<0.001, p<0.001, p = 0.01, respectively), whereas the maximum LAV parameters did not show significant differences.

**Table 3:** Functional associations with AF Burden score.

AF Burden score [patients]	1 [8]	2 [65]	3 [23]	4 [5]	p value
LAV_max [ml], median ± IQR	84.5 ± 35.6	103.5 ± 38.8	97.8 ± 44.6	117.9 ± 24.4	0.36
LAV_min [ml], mean ± SD	37.9 ± 14.3	52.3 ± 20.4	56.4 ± 24.9	99.3 ± 85.6	**<0.001**
LAV_preA [ml], median ± IQR	69.2 ± 32.9	79.5 ± 32.3	76.3 ± 47.7	98.8 ± 42.9	0.42
LAV_min2 [ml], median ± IQR	60.9 ± 32.8	74.9 ± 33.0	69.3 ± 43.3	96.8 ± 46.4	0.45
LAVi_max [ml/m2], median ± IQR	41.1 ± 10.7	50.9 ± 19.6	45.2 ± 22.0	55.8 ± 22.8	0.20
LAVi_min [ml/m2], mean ± SD	18.9 ± 5.9	25.7 ± 9.6	28.5 ± 12.5	51.1 ± 42.7	**<0.001**
LAVi_preA [ml/m2], mean ± SD	33.3 ± 6.2	39.4 ± 11.8	40.4 ± 12.5	59.6 ± 42.0	**0.01**
LAVi_min2 [ml/m2], mean ± SD	31.0 ± 7.2	37.0 ± 11.4	38.8 ± 12.4	58.1 ± 43.2	0.009
LAEF_total [%], mean ± SD	57.2 ± 10.3	49.8 ± 10.3	44.5 ± 10.9	34.9 ± 24.6	**0.002**
LAEF_active [%], mean ± SD	44.0 ± 11.0	36.2 ± 11.0	31.7 ± 11.8	20.8 ± 17.5	**0.003**
LAEF_passive [%], mean ± SD	24.2 ± 5.6	22.3 ± 7.8	18.7 ± 5.9	20.3 ± 15.4	0.19

LAEF–Left atrial emptying fraction, LAV–Left atrial volume, LAVi–Left atrial volume index

### Association of functional parameters with symptom severity & atrial fibrillation type

No significant differences was observed between EHRA score or AF type (paroxysmal or persistent AF) and any of the LA parameters (S2 and S3 Tables).

### Prediction of functional left atrial parameters

Multivariable linear regression models were computed for total, active and passive LAEF in relation to the classification of AF based (stroke risk (CHA_2_DS_2_VASc score), symptom severity (EHRA score), severity of AF Burden (type of AF and AFBS)) and CV risk factors. The results from univariable LRM can be found in the S4–S6 Tables.

#### Total, active and passive LAEF and AF risk factors

The mLRM was corrected for age, sex, BMI and showed that LAEF_total decreased per year of age by -0.49% (95%CI: -0.71, -0.27, p<0.001), in the presence of HF by -14.23% (95%CI: -23.82, -4.64, p = 0.004) and diagnosis of HT by -5.51% (95%CI: -9.80, -1.23), p = 0.01) (Table 4); excluded parameters can be found in the S7–S9 Tables.

**Table 4:** Multivariable linear regression analyses for predicting factors.

Parameter	Variable	B	95% CI	P value
Total LAEF	(Intercept)	75.98	57.27, 94.69	**<0.001**
	Age	-0.49	-0.71, -0.27	**<0.001**
	Male Sex	3.79	-1.62, 9.19	0.17
	BMI	-0.01	-0.53, 0.51	0.96
	Heart failure	-14.23	-23.82, -4.64	**0.004**
R^2^ adjusted: 0.32	Arterial hypertension	-5.51	-9.80, -1.23	**0.01**
Active LAEF	(Intercept)	61.82	39.43, 84.21	**<0.001**
	Age	-0.32	-0.58, -0.05	**0.02**
	Male Sex	2.80	-3.40, 8.99	0.37
	BMI	0.02	-0.55, 0.58	0.96
	AF Burden	-4.20	-7.88, -0.53	**0.026**
R^2^ adjusted: 0.22	Heart failure	-12.12	-23.61, -0.63	**0.039**
Passive LAEF	(Intercept)	18.10	-3.52, 39.72	0.10
	Age	-0.27	-0.45, -0.09	**0.003**
	Male Sex	2.28	-2.01, 6.57	0.29
	BMI	0.05	-0.34, 0.45	0.78
R^2^ adjusted: 0.14	LVEF	0.29	0.06, 0.53	**0.014**

95% CI– 95% Confidence interval, AF–Atrial fibrillation, BMI–Body mass index, LAEF–left atrial emptying fraction, LVEF–Left ventricular ejection fraction.

The corrected mLRM for LAEF_active showed a decrease per year of age by -0.32% (95% CI: -0.58, -0.05, p = 0.02), for each increase in AFBS category by -4.20% (95%CI: -7.88, -0.53, p = 0.026) and in the presence of HF by -12.12% (95% CI: -23.61, -0.63, p = 0.039) (Table 4). The significance of CHA_2_DS_2_VASc, quality of life, renal failure, HT and LVEF in the uLRM vanished in the multivariable approach (see Supplemental Material).

In contrast, the model for LAEF_passive only included a decrease by -0.27% (95%CI: -0.45, -0.09), p = 0.003) per year of age and an increase by 0.29% (95%CI: 0.06, 0.53), p = 0.014) per each percent of LVEF (Table 4). The effects of CHA_2_DS_2_VASc, HF and HT in the univariable models did not prevail in the multivariable model.

We note that the classical differentiation between paroxysmal and persistent AF (AF type) and EHRA score did not have an impact on any parameter for both uLRM or mLRM.

### Comprehensive analysis

The baseline characteristics from the comprehensive analysis of the entire cohort (n = 151 patients), including the patients in AF during MR acquisition, can be found in the Supplemental Material. Due to the 49 patients with AF during the MRI acquisition without active LA contraction, we could only investigate LAV_max, LAV_min, LAEF_total as well as LAVi_max and LAVi_min in all patients. In this cohort, we observed more patients with persistent AF and also overall higher AFBS (see S10 Table). AFBS and stroke risk based on CHA_2_DS_2_VASc were lower in patients with lower LAV_min (p<0.001, p = 0.01; respectively), lower LAVi_min (p<0.001, p<0.001; respectively) and higher total LAEF (p<0.001, p<0.001; respectively); S11 and S12 Tables. On opposite, LAV_max was not significantly different for AFBS and CHA_2_DS_2_VASc-based stroke risk. We also observed that patients with persistent AF had higher minimum and maximum volumes (both indexed and absolute) and a lower total LAEF; S13 Table. Lastly, EHRA score did not show significant differences; S14 Table.

## Discussion

In this study, we investigated LA functional parameters using a fully-automatic, 3D-based volumetric assessment of the whole cardiac cycle of the LA with a comprehensive clinical AF classification scheme. The main observations of our study were:

1. The application of the previously validated, CNN-based segmentation algorithm was feasible for 3D segmentation in all except three patients. Overall, automatic detection of fiducial points from the resulting volume-time curve was possible in 102/114 patients (92%), allowing to automatically differentiate between total, active and passive LAEF.
2. We identified strong association of the LA function with AFBS and stroke risk based on CHA_2_DS_2_VASc. In detail, LAEF_total and LAEF_active both showed a significant decline with increasing AFBS. In addition, patients with a lower total, active and passive LAEF presented with an increased stroke risk according to the CHA_2_DS_2_VASc. For all those associations, the increase of LAV_min rather than an increase of LAV_max (for LAEF_total) or LAV_preA (for LAEF_active) seemed to be the underlying mechanism. Results from the entire cohort including patients in AF during the MRI and therefore without active LA contraction did not differ substantially from these observations.
3. Multivariable regression analyses, corrected for age, BMI and sex, revealed relationships between total, active and passive LAEF with established AF risk factors. Especially higher age was an independent predictor of a reduction of all three LAEF parameters in our AF cohort. In addition, a higher AFBS was an independent predictor of reduced LAEF_active, HT was an independent predictor of reduced LAEF_total, and HF independently predicted reduced active and total LAEF.

### Applicability of the approach

LA size measured as maximum diameter in parasternal long axis by echocardiography at ventricular end-systole is an established parameter and was identified as predictor for AF in the Framingham Study [28]. For LAV assessment, superiority of cMRI over echocardiography was shown in the past while other techniques such as 3D mapping system also allow LA volumetry [29–31]. The vast majority of cMRI studies, however, used biplanar-based calculation for volumetric analysis of the LA with a focus on maximum (indexed) volume [16, 17, 32, 33]. Longer acquisition times for 3D coverage of the LA and the time-consuming manual LA segmentation might explain the limited number of cMRI studies analyzing LA function based on 3D datasets in the past [29, 34, 35]. Recently, there were reports of AI tools segmenting the LA on CINE series for biplane or short axis-based assessment but not for oblique-axial orientation [36–38]. To overcome this limitation, we recently validated a CNN-based algorithm for LA segmentation over the whole cardiac cycle. This algorithm has two elements, one for biplane-based LA segmentation and one for 3D-based LA segmentation on oblique-axial CINE series [21]; the latter was used in this study. Volume-time curves were generated from the segmentations and characteristic fiducial points automatically identified, which was possible in 102 patients. The reason for failed identification of fiducial points were rhythm irregularities (numerous extra-systoles) during image acquisition. Real-time CINE imaging could generally be an option to overcome arrhythmias, however, these were not acquired in this study. Due to low image resolution, volumetric assessment and therefore LAEF assessment could be compromised by real-time imaging.

Overall, automatic calculation of LAEF_total, LAEF_active and LAEF_passive could be achieved in 102 patients with sinus-rhythm during cMRI from a standard clinical protocol. This showed applicability of our comprehensive approach and represents an example of a fully automated, DL-supported workflow supporting a complex, cMRI-based analysis. However, in patients with AF during MRI acquisition, assessment of LAEF_active is not possible. Instead, our comprehensive analysis suggested that LAEF_total might serve as an alternative biomarker in this cohort.

### LA volumes and LAEF as measures for LA size and function and their clinical implications

Single time point-based analysis of LA volume is common in clinical practice since LA enlargement was linked to multiple CV diseases [39]. However, the potential superiority of LAEF as a functional parameter over a static LAV alone was proposed by Hoit who linked it to the importance of minimum LAV [39]. LAEF combines distinct measures of LAV_max, LAV_min and/or LAV_preA in one parameter and, therefore, strengthens the advantage of CINE analysis over single time point assessment. LAEF was already associated with silent strokes, HF and cardiomyopathies [15, 32, 33]. In patients with diagnosis of AF, Sievers et al. reported a mean total LAEF of 49.8% (in sinus rhythm) based on 3D assessment, respectively [34]; these results match our findings well (48.6%). Wandelt et al. performed manual 3D segmentations based on axial CINE series in an AF patient cohort. The reported mean values for LAEF_total, LAEF_active and LAEF_passive of 47.9%, 35.6% and 19.2%, respectively, were similar to our results (48.6%, 35.2% and 21.7%, respectively) [40].

#### LA function and AF burden

AF Burden is an important parameter to assess and classify AF patients [14]. In addition to the categorization in paroxysmal and persistent AF, we included AFBS, which characterizes AF Burden in more detail by combining frequency and duration of AF episodes as well as the number of cardioversions. These characteristics proved to be able to predict AF recurrence after first and repeated PVI better than the classic AF characterization [27]. In line with this observation, the AFBS, but not the conventional classification in paroxysmal and persistent AF, showed a significant relationship to active and total LAEF in this study. Accordingly, mLRM results showed a significant reduction of LAEF_active by -4.2% with each increase of AFBS category. These results were driven by a bigger difference of minimum volume rather than LAV_max for LAEF_total or LAV_preA for LAEF_active. The correlation of LAEF_active reduction with AFBS increase might be explained by the fact that a “healthier” (less remodeled) LA was able to actively pump more blood from the LA into the left ventricle at the end of ventricular diastole, resulting in a lower minimum LA volume. Other studies observed associations of decreased LAEF_active with non-obstructive, hypertrophic cardiomyopathy prior to LA enlargement and adverse effects and death in hypertensive patients [15, 41]. Our data suggested that LAEF_total and LAEF_active might play in addition an important role in assessment of AF patients, at least if they are in sinus rhythm during MRI.

#### LA function and stroke risk based on CHA_2_DS_2_VASc

Patients with AF suffer a 5-fold increased risk of stroke caused by thrombus formations in the LA and LA appendage [11]. The CHA_2_DS_2_VASc score as a measure of the stroke risk in AF patients showed another important association with LA function in our study: In patients with an increased risk for stroke (based on a CHA_2_DS_2_VASc ≥ 2), total, active and passive LAEF were significantly lower compared to low-risk patients (CHA_2_DS_2_VASc ≤ 1). This indicated that a reduced LA function was associated with an increased risk for stroke in AF patients. In fact, all 7 patients with a reported stroke in our cohort had a CHA_2_DS_2_VASc ≥ 2. This is in accordance with the current literature where a reduction of LAEF_total was linked to cerebrovascular events or in patients suffering a stroke [33, 42]. In accordance with our observation, Leung et al. stated that LA function could provide additional risk stratification for stroke in patient with a high CHA_2_DS_2_VASc score of ≥2 [43].

#### LA function and other AF risk factors

In a separate analysis, we furthermore identified significant relationships between established AF risk factors and the three LAEF parameters (LAEF_total, LAEF_active, LAEF_passive). In detail, the mLRM (corrected for age, BMI and sex), revealed a statistically significant, negative correlation of age with all three LAEF parameters. This is in accordance to the known importance of age as a risk factor for AF [44]. Arterial hypertension and HF which are other known, major AF risk factors, were also independent predictors of reduced total and active LAEF [39, 41]. Opposite, LVEF was (besides age) the only parameter to independently predictor a higher LAEF_passive. This in line with the fact that LAEF_passive is mainly determined by LV functionality [39, 45]. Of note, LAEF_passive cannot accurately assess the conduit function of the LA because blood can pass through the LA directly from the pulmonary veins without changing the current LA volume [39]. In summary, age, HT, HF and to a certain amount LVEF, are relevant, independent predictors of the LA function.

### Limitations

This was a single-center, retrospectively analyzed study from a prospective cohort, therefore generalizability of our results might be limited. AFBS is partially a subjective score, patients without or with milder symptoms could be underrepresented.

When we planned this study, we focused on LA volumes assessment in 3D. Therefore, we only have oblique-axial CINE series available in this cohort and could not compare our parameters to biplane assessment of LA function which was a tradeoff to allow for 3D image acquisition.

The applied, previously established CNN for LA segmentation was built in-house on imaging data from one vendor. While openly accessible, the transferability on imaging studies from other institutions cannot be guaranteed.

We investigated only patients whose volume-time curves had all fiducial points available. This restricted the generalizability of the results to patients in SR at the time of cMRI which might have caused a bias regarding patient selection, for example this could have limited the number of patients with persistent AF (28% of all patients). To address this limitation, we performed the comprehensive analysis of the entire cohort, including patients with AF during the acquisition. Furthermore, we investigated the risk for stroke based on the CHA_2_DS_2_VASc and not based on the clinical event of a stroke. The CHA_2_DS_2_VASc was also not homogenously distributed in our rather young patient cohort.

We did not perform continuous rhythm monitoring before the MRI; therefore, a reduced LA function might as well be a result of LA stunning due to previous spontaneous termination of AF. Furthermore, short paroxysmal episodes of AF could have happened during MRI and could have led to a missing atrial contraction, resulting in exclusion of these patients. Finally, an adjustment for multiple testing was not performed due to the exploratory nature of the comparison.

## Conclusions

Our study showed that the fully-automatic characterization of LA function from 3D-based CINE cMRI is feasible in a clinical cohort of patients with diagnosis of AF. It revealed significant associations between LA functional parameters, especially active LAEF, with increased stroke risk (based on the CHA_2_DS_2_VASc score) and the severity of the AF Burden. This indicates potential usefulness of active LAEF as an imaging biomarker, though its effect on clinical endpoints such as recurrence of AF, hospitalization, stroke, or mortality, requires evaluation in further studies.

## Supporting information

S1 Table Functional associations with CHA_2_DS_2_VASc. LAEF parameters were significantly different between the CHA_2_DS_2_VASc categories.
(DOCX)

S2 Table Functional associations with EHRA score. No significant differences were seen between volumetric or functional parameters.
(DOCX)

S3 Table Functional associations with AF type. No significant differences were seen between volumetric or functional parameters.
(DOCX)

S4 Table Total LAEF—Excluded variables from multivariable regression analysis. (DOCX)

S5 Table Active LAEF—Excluded variables from multivariable regression analysis. (DOCX)

S6 Table Passive LAEF—Excluded variables from multivariable regression analysis. (DOCX)

S7 Table Univariable linear regression analyses for total LAEF. (DOCX)

S8 Table Univariable linear regression analyses for active LAEF. (DOCX)

S9 Table Univariable linear regression analyses for passive LAEF. (DOCX)

S10 Table Comprehensive analysis of the entire cohort. Baseline characteristics.
(DOCX)

S11 Table Comprehensive analysis, AF Burden score. Absolute and indexed minimum LA volume and LAEF_total were significantly different between groups while LAV_max was not.
(DOCX)

S12 Table Comprehensive analysis, CHA_2_DS_2_VASc based stroke risk. Absolute and indexed minimum LA volume and LAEF_total were significantly different between groups while LAV_max was not.
(DOCX)

S13 Table Comprehensive analysis, AF type. LA volumes (minimum and maximum) were higher and LAEF_total lower in patients with persistent AF.
(DOCX)

S14 Table Comprehensive analysis, EHRA score. We did not find significant differences between EHRA Score and LA measures.
(DOCX)

S1 Data Minimal data set. (XLSX)

## Abbreviations

2D: 2-dimensional
3D: 3-dimensional
AF: Atrial fibrillation
AFBS: AF Burden Score
AI: Artificial intelligence
BMI: Body mass index
BSA: Body surface area
cMRI: Cardiac Magnetic Resonance Imaging
CNN: Convolutional Neural Network
CV: Cardiovascular
DL: Deep learning
ECG: Electrocardiogram
EHRA: European Heart Rhythm Association
HF: Heart failure
HT: Arterial hypertension
LA: Left atrium
LAEF: Left atrial emptying fraction
LAEF_active: Active LAEF
LAEF_passive: Passive LAEF
LAEF_total: Total LAEF
LAV: Left atrial volume
LAV_max: Maximum left atrial volume
LAV_min: Minimum left atrial volume
LAV_min2: Left atrial volume, local minimum
LAV_preA: Left atrial volume before atrial contraction
LAVi: Left atrial volume index
LAVi_max: Indexed maximum left atrial volume
LAVi_min: Minimum left atrial volume index
LAVi_min2: Left atrial volume indexed, local minimum
LAVi_preA: Left atrial volume index before atrial contraction
LVEF: Left ventricular ejection fraction
mLRM: Multivariable linear regression model
SD: Standard deviation
SR: Sinus rhythm
TE: Echo time
uLRM: Univariable linear regression model
