# Supplementary material for: Associations between fully-automated, 3D-based functional analysis of the left atrium and classification schemes in atrial fibrillation
Source: PLoS One. 2022 Aug 15;17(8):e0272011. doi: 10.1371/journal.pone.0272011 (PMC9377598; doi:10.1371/journal.pone.0272011)
Supplement: S1 Table — LAEF parameters were significantly different between the CHA2DS2VASc categories. (DOCX) [file pone.0272011.s001.docx]

Supplemental Information

| **S1 Table: Functional associations with CHA_2_DS_2_VASC** | | | | | | | | | |
| --- | --- | --- | --- | --- | --- | --- | --- | --- | --- |
| CHA2DS2VASC [patients] | 0 [n=23] | 1  [n=31] | 2  [n=29] | 3  [n=11] | 4  [n=6] | 5  [n=1] | 6  [n=0] | p value |  |
| LAV_max [ml] | 99.8 (26.5) | 92.4 (46.4) | 104.8 (30.1) | 104.1 (56.6) | 111.2 (12.1) | 94 | n/a | 0.78 |  |
| LAV_min [ml] | 42.6 (12.4) | 45.0 (26.8) | 55.3 (23.9) | 62.9 (45.1) | 62.5 (27.8) | 48.7 | n/a | 0.12 |  |
| LAV_preA [ml] | 71.0 (16.7) | 69.3 (38.9) | 82.0 (25.7) | 86.3 (51.3) | 83.1 (17.9) | 83 | n/a | 0.27 |  |
| LAV_min2 [ml] | 67.1 (18.3) | 61.6 (33.5) | 81.4 (30.3) | 85.1 (46.0) | 80.7 (14.0 | 80.8 | n/a | 0.11 |  |
| LAVi_max [ml/m^2^] | 46.7 (13.4) | 46.6 (18.8) | 52.5 (20.2) | 61.9 (29.1) | 55.7 (8.9) | 49.4 | n/a | 0.63 |  |
| LAVi_min [ml/m^2^] | 20.4 (6.5) | 22.8 (10.8) | 27.7 (16.2) | 31.7 (26.2) | 32.3 (15.1) | 25.6 | n/a | 0.06 |  |
| LAVi_preA [ml/m^2^] | 35.0 (9.3) | 35.5 (15.6) | 40.9 (16.7) | 51.3 (27.7) | 42.8 (7.8) | 43.6 | n/a | 0.14 |  |
| LAVi_min2 [ml/m^2^] | 32.5 (11.6) | 32.1 (15.5) | 37.9 (16.9) | 48.7 (27.0) | 41.7 (7.9) | 42.5 | n/a | **<0.05** |  |
| LAEF_total [%] | 55.8 (12.2) | 53.7 (10.3) | 46.1 (7.7) | 40.5 (19.9) | 43.0 (18.9) | 48.2 | n/a | **0.002** |  |
| LAEF_active [%] | 42.1 (14.5) | 37.4 (8.0) | 30.5 (13.3) | 32.8 (20.7) | 26.2 (20.9) | 41.3 | n/a | **0.04** |  |
| LAEF_passive [%] | 24.6 (8.8) | 24.3 (8.6) | 18.7 (8.3) | 17.1 (3.8) | 18.7 (8.4) | 11.7 | n/a | **0.001** |  |

All parameters represent median (IQR)
