# Supplementary material for: Associations between fully-automated, 3D-based functional analysis of the left atrium and classification schemes in atrial fibrillation
Source: PLoS One. 2022 Aug 15;17(8):e0272011. doi: 10.1371/journal.pone.0272011 (PMC9377598; doi:10.1371/journal.pone.0272011)
Supplement: S2 Table — No significant differences were seen between volumetric or functional parameters. (DOCX) [file pone.0272011.s002.docx]

Supplemental Information

| **S2 Table: Functional associations with EHRA score** | | | | | |
| --- | --- | --- | --- | --- | --- |
| Score  [patients] | 1  [n=8] | 2  [n=57] | 3  [n=30] | 4  [n=1] | p value |
| LAV_max [ml], median ± IQR | 87.6±35.0 | 103.5±35.4 | 92.5±53.0 | 121.2 | 0.32 |
| LAV_min [ml],  median ± IQR | 34.5±22.7 | 49.7±24.3 | 47.9±30.8 | 75.7 | 0.36 |
| LAV_preA [ml],  median ± IQR | 67.4±27.0 | 75.6±29.7 | 76.2±43.4 | 102.6 | 0.53 |
| LAV_min2 [ml],  median ± IQR | 66.0±25.7 | 69.3±27.6 | 72.1±42.6 | 96.0 | 0.67 |
| LAVi_max [ml/m2],  median ± IQR | 41.9±16.1 | 50.3±22.1 | 45.6±18.8 | 61.2 | 0.37 |
| LAVi_min [ml/m2],  median ± IQR | 17.1±11.7 | 23.2±12.5 | 24.6±12.7 | 38.2 | 0.38 |
| LAVi_preA [ml/m2],  median ± IQR | 32.3±13.0 | 37.8±16.7 | 35.0±17.7 | 51.8 | 0.59 |
| LAVi_min2 [ml/m2],  median ± IQR | 31.6±12.4 | 35.5±16.6 | 33.1±18.2 | 48.5 | 0.74 |
| LAEF_total [%],  median ± IQR | 56.1±12.5 | 50.8±13.0 | 48.3±7.9 | 37.6 | 0.34 |
| LAEF_active [%],  median ± IQR | 41.7±13.6 | 36.5±14.9 | 37.4±11.2 | 26.2 | 0.35 |
| LAEF_passive [%],  median ± IQR | 18.9±4.7 | 23.0±9.4 | 19.1±8.3 | 15.3 | 0.07 |
