# Supplementary material for: Associations between fully-automated, 3D-based functional analysis of the left atrium and classification schemes in atrial fibrillation
Source: PLoS One. 2022 Aug 15;17(8):e0272011. doi: 10.1371/journal.pone.0272011 (PMC9377598; doi:10.1371/journal.pone.0272011)
Supplement: S3 Table — No significant differences were seen between volumetric or functional parameters. (DOCX) [file pone.0272011.s003.docx]

Supplemental Information

| **S3 Table: Functional associations with AF type** | | | |
| --- | --- | --- | --- |
| Parameter, mean ± SD  [Patients] | Paroxysmal  [73] | Persistent  [29] | p value |
| LAV_max [ml] | 99.0±28.9 | 111.4±44.2 | 0.1 |
| LAV_min [ml] | 50.7±20.0 | 63.0±43.0 | >0.05 |
| LAV_preA [ml] | 77.5±24.5 | 89.0±42.4 | 0.09 |
| LAV_min2 [ml] | 73.2±23.5 | 84.0±43.1 | 0.11 |
| LAVi_max [ml/m2] | 49.6±13.2 | 54.2±21.6 | 0.19 |
| LAVi_min [ml/m2] | 25.4±9.8 | 30.9±21.7 | 0.08 |
| LAVi_preA [ml/m2] | 38.8±11.2 | 43.4±21.1 | 0.16 |
| LAVi_min2 [ml/m2] | 36.7±10.9 | 41.1±21.6 | 0.18 |
| LAEF_total [%] | 49.6±10.6 | 46.1±15.2 | 0.2 |
| LAEF_active [%] | 36.1±11.5 | 32.8±13.8 | 0.23 |
| LAEF_passive [%] | 22.1±7.4 | 20.6±9.0 | 0.4 |
