# Supplementary material for: Associations between fully-automated, 3D-based functional analysis of the left atrium and classification schemes in atrial fibrillation
Source: PLoS One. 2022 Aug 15;17(8):e0272011. doi: 10.1371/journal.pone.0272011 (PMC9377598; doi:10.1371/journal.pone.0272011)
Supplement: S4 Table — (DOCX) [file pone.0272011.s004.docx]

Supplemental Information

| **S4 Table. Univariable regression analyses for total LAEF** | | | | | | |
| --- | --- | --- | --- | --- | --- | --- |
| Variable | B | β | t | p | 95% CI | |
| Age | -.532 | -.391 | -4.246 | **.000** | -.781 | -.283 |
| Sex | 4.572 | .144 | 1.459 | .148 | -1.644 | 10.788 |
| BMI | -.267 | -.088 | -.887 | .377 | -.865 | .330 |
| AF Burden | -6.633 | -.368 | -3.936 | **.000** | -9.977 | -3.289 |
| AF type | -1.709 | -.128 | -1.288 | .201 | -4.342 | .924 |
| CHA_2_DS_2_VASC | -3.526 | -.346 | -3.693 | **.000** | -5.421 | -1.632 |
| Increased stroke risk | -6.461 | -.267 | -2.453 | **.016** | -11.688 | -1.234 |
| Quality of life | .134 | .192 | 1.835 | .070 | -.011 | .278 |
| EHRA score | -2.562 | -.139 | -1.362 | .177 | -6.298 | 1.174 |
| Heart failure | -23.485 | -.460 | -5.152 | **.000** | -32.530 | -14.441 |
| Arterial hypertension | -7.285 | -.301 | -3.140 | **.002** | -11.888 | -2.682 |
| Diabetes | -5.676 | -.111 | -1.113 | .269 | -15.798 | 4.446 |
| Renal failure | -8.705 | -.183 | -1.853 | .067 | -18.024 | .614 |
| LVEF | .495 | .320 | 3.252 | **.002** | .193 | .797 |
