# Supplementary material for: Associations between fully-automated, 3D-based functional analysis of the left atrium and classification schemes in atrial fibrillation
Source: PLoS One. 2022 Aug 15;17(8):e0272011. doi: 10.1371/journal.pone.0272011 (PMC9377598; doi:10.1371/journal.pone.0272011)
Supplement: S5 Table — (DOCX) [file pone.0272011.s005.docx]

Supplemental Information

| **S5 Table. Univariable regression analyses for active LAEF** | | | | | | |
| --- | --- | --- | --- | --- | --- | --- |
| Variable | B | β | t | p | 95% CI | |
| Age | -.387 | -.283 | -2.947 | **.004** | -.648 | -.126 |
| Sex | 4.617 | .145 | 1.465 | .146 | -1.635 | 10.868 |
| BMI | .028 | .009 | .092 | .927 | -.575 | .631 |
| AF Burden | -6.475 | -.356 | -3.786 | **.000** | -9.868 | -3.082 |
| AF type | -1.620 | -.120 | -1.212 | .228 | -4.270 | 1.031 |
| CHA_2_DS_2_VASC | -2.782 | -.272 | -2.824 | **.006** | -4.737 | -.828 |
| Increased stroke risk | -5.868 | -.241 | -2.098 | **.038** | -11.418 | -.318 |
| Quality of life | .162 | .222 | 2.135 | **.036** | .011 | .313 |
| EHRA score | -1.696 | -.089 | -.863 | .390 | -5.599 | 2.206 |
| Heart failure | -21.173 | -.415 | -4.544 | **.000** | -30.418 | -11.928 |
| Arterial hypertension | -5.641 | -.234 | -2.390 | **.019** | -10.325 | -.958 |
| Diabetes | -4.261 | -.084 | -.835 | .406 | -14.389 | 5.867 |
| Renal failure | -6.569 | -.138 | -1.391 | .167 | -15.937 | 2.799 |
| LVEF | .435 | .281 | 2.825 | **.006** | .129 | .741 |
