# Supplementary material for: Associations between fully-automated, 3D-based functional analysis of the left atrium and classification schemes in atrial fibrillation
Source: PLoS One. 2022 Aug 15;17(8):e0272011. doi: 10.1371/journal.pone.0272011 (PMC9377598; doi:10.1371/journal.pone.0272011)
Supplement: S6 Table — (DOCX) [file pone.0272011.s006.docx]

Supplemental Information

| **S6 Table. Univariable regression analyses for passive LAEF** | | | | | | |
| --- | --- | --- | --- | --- | --- | --- |
| Variable | B | β | t | p | 95% CI | |
| Age | -.322 | -.364 | -3.907 | **.000** | -.485 | -.158 |
| Sex | 1.234 | .060 | .601 | .549 | -2.838 | 5.306 |
| BMI | -.195 | -.099 | -.996 | .322 | -.582 | .193 |
| AF Burden | -2.233 | -.191 | -1.934 | **.056** | -4.525 | .058 |
| AF type | -.732 | -.084 | -.846 | .400 | -2.450 | .985 |
| CHA_2_DS_2_VASC | -2.175 | -.329 | -3.484 | **.001** | -3.413 | -.936 |
| Increased stroke risk | -4.555 | -.290 | -2.620 | **.010** | -8.006 | -1.104 |
| Quality of life | -.009 | -.019 | -.176 | .861 | -.109 | .092 |
| EHRA score | -.942 | -.076 | -.739 | .462 | -3.474 | 1.589 |
| Heart failure | -8.071 | -.244 | -2.500 | **.014** | -14.478 | -1.665 |
| Arterial hypertension | -3.091 | -.197 | -1.999 | **.048** | -6.161 | -.022 |
| Diabetes | -4.464 | -.135 | -1.353 | .179 | -11.010 | 2.081 |
| Renal failure | -5.540 | -.180 | -1.818 | .072 | -11.588 | .508 |
| LVEF | .216 | .215 | 2.120 | **.037** | .014 | .418 |
