# Supplementary material for: Associations between fully-automated, 3D-based functional analysis of the left atrium and classification schemes in atrial fibrillation
Source: PLoS One. 2022 Aug 15;17(8):e0272011. doi: 10.1371/journal.pone.0272011 (PMC9377598; doi:10.1371/journal.pone.0272011)
Supplement: S7 Table — (DOCX) [file pone.0272011.s007.docx]

Supplemental Information

| **S7 Table. Total LAEF – excluded variables from multivariable regression analysis** | | | |
| --- | --- | --- | --- |
|  | B | t | p |
|  |  |  |  |
| LVEF | .190 | 1.858 | .067 |
| AF Burden | -.200 | -1.990 | .050 |
| CHA_2_DS_2_VASC | .091 | .639 | .525 |
| Increased stroke risk | -.097 | -.761 | .449 |
| Quality of life | .116 | 1.182 | .241 |
| EHRA score | -.007 | -.073 | .942 |
| Diabetes | -.148 | -1.464 | .148 |
| Renal failure | -.038 | -.364 | .717 |
| AF type | -.064 | -.643 | .522 |
