# Supplementary material for: Associations between fully-automated, 3D-based functional analysis of the left atrium and classification schemes in atrial fibrillation
Source: PLoS One. 2022 Aug 15;17(8):e0272011. doi: 10.1371/journal.pone.0272011 (PMC9377598; doi:10.1371/journal.pone.0272011)
Supplement: S8 Table — (DOCX) [file pone.0272011.s008.docx]

Supplemental Information

| **S8 Table. Active LAEF – excluded variables from multivariable regression analysis** | | | |
| --- | --- | --- | --- |
|  | B | t | p |
|  |  |  |  |
| LVEF | .122 | 1.087 | .281 |
| CHA_2_DS_2_VASC | .019 | .131 | .896 |
| Increased stroke risk | -.117 | -.893 | .375 |
| Quality of life | .173 | 1.651 | .103 |
| EHRA score | -.024 | -.232 | .817 |
| Arterial hypertension | -.178 | -1.680 | .097 |
| Diabetes | -.130 | -1.186 | .240 |
| Renal failure | -.005 | -.045 | .964 |
| AF type | .034 | .287 | .775 |
