# Supplementary material for: Associations between fully-automated, 3D-based functional analysis of the left atrium and classification schemes in atrial fibrillation
Source: PLoS One. 2022 Aug 15;17(8):e0272011. doi: 10.1371/journal.pone.0272011 (PMC9377598; doi:10.1371/journal.pone.0272011)
Supplement: S9 Table — (DOCX) [file pone.0272011.s009.docx]

Supplemental Information

| **S9 Table. Passive LAEF– excluded variables from multivariable regression analysis** | | | |
| --- | --- | --- | --- |
|  | B | T | p |
|  |  |  |  |
| AF Burden | -.075 | -.687 | .494 |
| CHA_2_DS_2_VASC | -.221 | -1.529 | .131 |
| Increased stroke risk | -.241 | -1.719 | .09 |
| Quality of life | -.125 | -1.134 | .261 |
| EHRA score | -.004 | -.039 | .969 |
| Heart failure | -.007 | -.063 | .950 |
| Arterial hypertension | -.102 | -.924 | .359 |
| Diabetes | -.117 | -1.015 | .314 |
| Renal failure | -.119 | -1.085 | .282 |
| AF type | -.090 | -.809 | .421 |
