# Supplementary material for: Associations between fully-automated, 3D-based functional analysis of the left atrium and classification schemes in atrial fibrillation
Source: PLoS One. 2022 Aug 15;17(8):e0272011. doi: 10.1371/journal.pone.0272011 (PMC9377598; doi:10.1371/journal.pone.0272011)
Supplement: S10 Table — Baseline characteristics. (DOCX) [file pone.0272011.s010.docx]

Supplemental Information

| **S10 Table** | Main cohort  (Patients w sinus rhythm during MRI, n=102) | Total cohort  (comprehensive analysis, n=151) |
| --- | --- | --- |
| AF Burden Score |  |  |
| 1 | 8 (7.8%) | 8 (5.3%) |
| 2 | 65 (63.7%) | 77 (51.0%) |
| 3 | 23 (22.5%) | 40 (26.5%) |
| 4 | 5 (4.9%) | 25 (15.6%) |
| Type AF |  |  |
| Paroxysmal | 73 (71.6%) | 87 (57.6%) |
| Persistent | 29 (28.4%) | 64 (42.4%) |
| EHRA Score |  |  |
| I | 8 (8%) | 11 (7.9%) |
| II | 57 (56%) | 80 (57.1%) |
| III | 30 (29%) | 46 (32.9%) |
| IV | 1 (1%) | 3 (2.1%) |
| CHA_2_DS_2_VASc-based stroke risk |  |  |
| Low stroke risk  (CHA_2_DS_2_VASc ≤ 1) | 55 (53.9%) | 82 (54.3%) |
| Increased stroke risk (CHA_2_DS_2_VASc ≥ 2) | 47 (46.1%) | 69 (45.7%) |
