# Supplementary material for: Associations between fully-automated, 3D-based functional analysis of the left atrium and classification schemes in atrial fibrillation
Source: PLoS One. 2022 Aug 15;17(8):e0272011. doi: 10.1371/journal.pone.0272011 (PMC9377598; doi:10.1371/journal.pone.0272011)
Supplement: S11 Table — Absolute and indexed minimum LA volume and LAEF_total were significantly different between groups while LAV_max was not. (DOCX) [file pone.0272011.s011.docx]

Supplemental Information

| **S1 Table 11** | **AF Burden Score** | | | | |
| --- | --- | --- | --- | --- | --- |
| AF Burden Score | 1 | 2 | 3 | 4 | p value |
| total cohort (n=151) |  |  |  |  |  |
| LAV_max [ml] | 84.5±35.6 | 104.1±39.2 | 106.4±48.1 | 118.1±32.6 | 0.068 |
| LAV_min [ml] | 37.9±16.7 | 53.4±34.5 | 69.4±51.7 | 94.2±45.0 | **0.001** |
| LAEF_total [ml] | 57.2±5.5 | 48.2±19.8 | 34.3±27.2 | 21.6±19.8 | **0.001** |
| LAVi_max [ml] | 41.1±10.7 | 52.0±18.7 | 52.0±24.8 | 59.4±22.8 | **0.049** |
| LAVi_min [ml] | 18.7±3.6 | 26.4±16.2 | 32.9±25.3 | 49.0±24.1 | **0.001** |
