# Supplementary material for: Associations between fully-automated, 3D-based functional analysis of the left atrium and classification schemes in atrial fibrillation
Source: PLoS One. 2022 Aug 15;17(8):e0272011. doi: 10.1371/journal.pone.0272011 (PMC9377598; doi:10.1371/journal.pone.0272011)
Supplement: S12 Table — Absolute and indexed minimum LA volume and LAEF_total were significantly different between groups while LAV_max was not. (DOCX) [file pone.0272011.s012.docx]

Supplemental Information

| **S12 Table** | **CHA_2_DS_2_VASc-based stroke risk** | | |
| --- | --- | --- | --- |
| stroke risk | low_risk | increased_risk | p value |
| total cohort (n=151) |  |  |  |
| LAV_max [ml] | 105.3±38.1 | 110.7±33.8 | 0.362 |
| LAV_min [ml] | 50.1±38.9 | 67.9±45.5 | **0.011** |
| LAEF_total [ml] | 47.9±27.5 | 39.3±28.6 | **0.001** |
| LAVi_max [ml] | 48.7±18.1 | 55.6±19.2 | **0.028** |
| LAVi_min [ml] | 24.5±17.3 | 35.8±22.8 | **0.001** |
