# Supplementary material for: Associations between fully-automated, 3D-based functional analysis of the left atrium and classification schemes in atrial fibrillation
Source: PLoS One. 2022 Aug 15;17(8):e0272011. doi: 10.1371/journal.pone.0272011 (PMC9377598; doi:10.1371/journal.pone.0272011)
Supplement: S13 Table — LA volumes (minimum and maximum) were higher and LAEF_total lower in patients with persistent AF. (DOCX) [file pone.0272011.s013.docx]

Supplemental Information

| **S13 Table** | **AF Type** | | |
| --- | --- | --- | --- |
| AF Type | Paroxysmal AF | Persistent AF | p value |
| total cohort (n=151) |  |  |  |
| LAV_max [ml] | 102.3±37.1 | 115.5±45.8 | **0.002** |
| LAV_min [ml] | 51.6±30.5 | 84.2±60.0 | **0.001** |
| LAEF_total [ml] | 47.8±20.0 | 25.2±31.0 | **0.001** |
| LAVi_max [ml] | 49.7±18.0 | 55.6±24.6 | **0.006** |
| LAVi_min [ml] | 25.7±15.9 | 39.5±30.9 | **0.001** |
