# Supplementary material for: Associations between fully-automated, 3D-based functional analysis of the left atrium and classification schemes in atrial fibrillation
Source: PLoS One. 2022 Aug 15;17(8):e0272011. doi: 10.1371/journal.pone.0272011 (PMC9377598; doi:10.1371/journal.pone.0272011)
Supplement: S14 Table — We did not find significant differences between EHRA Score and LA measures. (DOCX) [file pone.0272011.s014.docx]

Supplemental Information

| **S14 Table** | **EHRA Score** | | | | |
| --- | --- | --- | --- | --- | --- |
| EHRA Score | I | II | III | IV | p value |
| total cohort (n=151) |  |  |  |  |  |
| LAV_max [ml] | 94.1±28.3 | 104.9±40.9 | 102.5±45.1 | 121.2±12.9 | 0.584 |
| LAV_min [ml] | 50.1±48.2 | 56.6±37.7 | 60.2±49.0 | 84.6±10.0 | 0.306 |
| LAEF_total [ml] | 50.0±30.7 | 45.2±26.8 | 44.8±28.4 | 26.3±9.4 | 0.201 |
| LAVi_max [ml] | 43.3±20.9 | 50.9±23.5 | 53.3±20.0 | 61.2±6.3 | 0.769 |
| LAVi_min [ml] | 26.6±25.0 | 27.3±19.7 | 31.6±23.6 | 40.1±3.7 | 0.247 |
